# Electrochemical Lithium Extraction with Gas Flushing of Porous Electrodes

**DOI:** 10.3390/nano13091471

**Published:** 2023-04-26

**Authors:** Shengyao Wang, Xuyu Yu, Xuejiao Hu

**Affiliations:** MOE Key Laboratory of Hydraulic Machinery Transients, School of Power and Mechanical Engineering, Wuhan University, Wuhan 430072, China; wangshengyao@whu.edu.cn (S.W.);

**Keywords:** electrochemical lithium extraction, gas flush, flow-through, porous electrodes

## Abstract

Electrochemical extraction of lithium from seawater/brine is receiving more and more attention because of its environment-friendly and energy-saving features. In this work, an electrochemical lithium extraction system with gas flushing of porous electrodes is proposed. We verified that the operation of multiple gas washes can significantly reduce the consumption of ultrapure water during the solution exchange and save the time required for the continuous running of the system. The water consumption of multiple gas flush operations is only 1/60 of that of a normal single flush to obtain a purity close to 100% in the recovery solution. By comparing the ion concentration distribution on the electrode surface in flow-through and flow-by-flow modes, we demonstrate that the flow-through mode performs better. We also verified the lithium extraction performance of the whole system, achieving a purity close to 100% and average energy consumption of 0.732 kWh∙kg^−1^ in each cycle from the source solution of the simulated Atacama salt lake water. These results provide a feasible approach for the large-scale operation of electrochemical lithium extraction from seawater/brine.

## 1. Introduction

With the large-scale application of lithium-ion batteries, the demand for lithium resources has grown significantly in recent years [1,2]. Global lithium ore reserves are gradually decreasing and can no longer meet the future market demand [3,4,5]. More than 60% of the global lithium resources exist in salt lakes and seawater, with a much larger total content than lithium ores [6]. It is promising to develop efficient lithium recovery technology to extract lithium from aqueous solutions [7,8,9,10]. The electrochemical method is considered the most promising one due to its excellent lithium selectivity, high insertion capacity, low energy consumption, high reversibility, and eco-friendliness [11,12,13,14].

Electrochemical lithium extraction technology has been intensively studied and developed in the last decade. Its working principle consists of four steps [15,16,17,18,19]. In the first step, a negative current is applied to the cathode, and lithium ions are captured from the feed solution through the intercalation of lithium-selective materials. In the second step, the brine is exchanged with the recovery solution. In the third step, a reverse current is applied to release the lithium ions trapped in the electrode, and then a high-purity lithium recovery solution is obtained. In the fourth step, new brine is used to replace the recovery solution for the next cycle.

Most of the previous research on electrochemical lithium extraction focused on improving electrode materials [20,21,22,23,24] and optimizing charging and discharging conditions [25,26,27]. The researches on electrochemical lithium extraction operating system are very limited. Zhao et al. developed a semi-continuous flow NMMO/AC hybrid supercapacitor. To enrich the lithium concentration, they repeatedly pumped the same brine and recovery solution into the system and obtained a purity of 97.2% Li^+^ from the simulated brine [28]. Kim et al. proposed a sustainable redox-mediated lithium recovery system using a redox couple of ferri-/ferrocyanide with adsorbent (λ-MnO_2_). It enriched lithium up to 37 mM Li^+^ from the feed solution with an equimolar concentration of 5 mM Li^+^ and 5 mM Na^+^ [29]. These studies obtained lithium recovery solution with higher purity and concentration by repeated adsorption process and continuous long-time adsorption through system operation design but did not establish a complete lithium extraction operation system. In our work, we built a flow-type electrochemical system with a porous electrode that contains all the operational steps. This system can run automatically and continuously, greatly simplifying the operational complexity of the lithium extraction process.

In addition to the system construction, the issue of ultrapure water consumption is also a practical obstacle to large-scale applications of electrochemical lithium extraction, although the vast majority of lithium extraction studies took it as a basic operation and didn’t mention it. In preparation for the lithium-ion release, the brine solution needs to be replaced with a recovery solution after the electrode captures the lithium ions. This process involves taking the adsorption electrode out of the reaction cell and rinsing the electrode and the device with a large amount of ultrapure water. Aiming to avoid the residual high sodium concentration brine solution will reduce the purity of the final recovery solution. Palagonia et al. mentioned the flushing process in their lithium extraction reactor and used 50 mL of KCL solution continuously pumped into the reactor for cleaning [30], even though the actual reactor volume was less than 1 mL. In order to give an optimized method that takes into account both the purity of the recovery solution and the consumption of ultrapure water, we introduce a gas flushing operation into the system and perform detailed experiments and theoretical calculations.

Moreover, Electrochemical reactors for the extraction of lithium have been described in recent years, using both flow-through and flow-by configurations [31]. Romero et al. reported a flow-by-cation-exchange electrochemical reactor consisting of two Li_1−x_Mn_2_O_4_/LiMn_2_O_4_ electrodes for the capture and release, respectively, of lithium from the brine into a recovery electrolyte [32]. Palagonia et al. reported a flow-through lithium extraction device with an LMO/NiHCF system, which yielded a recovery solution with a concentration of 100 mM at 94% purity after nine cycles [30]. However, there is no clear comparison of the lithium extraction performance of the two flow modes. To provide an idea for the selection of flow mode for lithium extraction reactors, we compared the mass transfer performance of flow-by and flow-through lithium extraction reactors by simulating.

In this work, a flow electrochemical lithium extraction system with gas flushing operation is proposed, in which the water consumption is only 1/60 of that of a normal single flush to obtain a purity close to 100% in the recovery solution. The mass transfer performance of flow-through and flow-by models was investigated by COMSOL simulation, showing that the flow-through mode has a higher lithium concentration on the electrode surface. The lithium extraction performance of the system was tested in simulated salt lake water at Atacama and a lithium extraction with a purity close to 100% (4.4% in the original solution) and an average energy consumption of 0.732 kWh∙kg^−1^ was achieved. These results demonstrate the potential of electrochemical lithium extraction technology for practical applications and the feasibility of using gas flush operation optimization and flow-through convection optimization.

## 2. Experiment

### 2.1. Gas Flushing Electrochemical Lithium Extraction System

**Figure 1:**
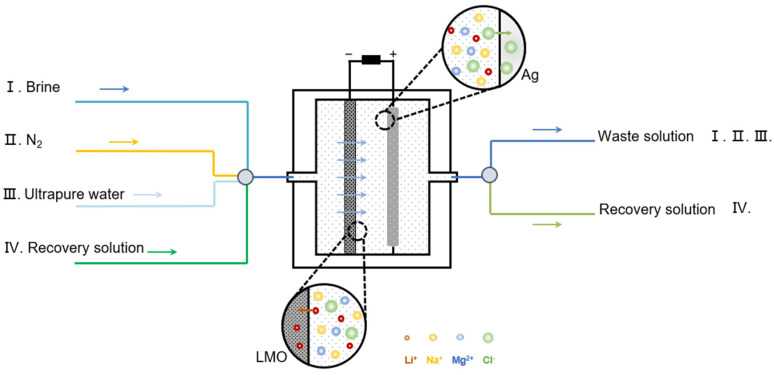
Schematic of gas flushing electrochemical lithium extraction system.

Figure 1 shows the schematic of the lithium extraction system. It includes a power supply, reaction chamber (porous lithium adsorption electrode, counter electrode), brine solution circuit, nitrogen circuit, ultrapure water circuit, and recovery solution circuit. The whole device is well-sealed. Pumps and valves can be controlled by a computer to control the solution or gas pumped into each circuit. Compared with the traditional electrochemical reaction cell, the whole system does not need to disassemble the electrodes during the reaction process and can run continuously, making the operation process extremely convenient.

In the reaction chamber, LiMn_2_O_4_ (LMO) spinel was selected as the Li^+^ adsorption electrode material. LMO is a common material for lithium extraction due to its good stability, low cost, high potential, and environmental friendliness. The spatial structure of the lithium manganate crystal is a three-dimensional tunnel structure. The ionic radius of Li^+^ coincides with the tetrahedral site of the spinel structure, enabling it to insert LMO. Other ions such as Na^+^, K^+^, and Ca^2+^ cannot be inserted due to their larger ionic radii. Although Mg^2+^ has a similar ionic radius to Li^+^, its hydration-free energy is four times that of Li^+^, resulting in greater insertion obstruction. Therefore, LMO can be used as a working electrode for the electrochemical extraction of lithium because of its high selectivity to lithium.

The operation of the gas-washing lithium extraction system is as follows. Stage (I), passing seawater/salt lake water solution into the device, apply negative current to the LMO electrode. Li^+^ ions are inserted in the cubic spinel structure, and the Ag electrode accordingly captures Cl^−^ to generate AgCl. Stage (II), passing N_2_, gas flush out the extracted source solution in the reaction chamber and flush out the residual solution inside the porous electrode. Stage (III), passing a small amount of ultrapure water to rinse the whole device and gas flush out the waste solution. Stage (IV), passing the recovery solution (generally LiCl solution) into the device, the charging process is carried out. Li^+^ in the LMO is released and AgCl is reduced to Ag. Gas flush the solution out of the chamber to obtain a high-purity lithium recovery solution.

**Figure 2:**
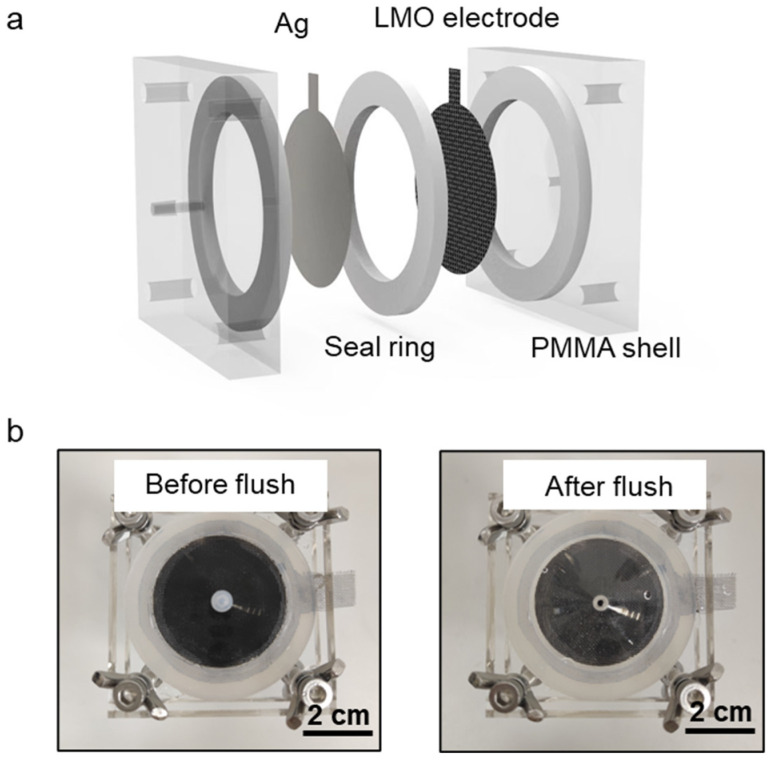
Electrochemical cell. (**a**) Schematic of the electrochemical cell. (**b**) Pictures of the electrochemical cell before and after gas flush.

The scheme of the electrochemical cell is shown in Figure 2a. It consists of an acrylic housing, silver sheet electrodes, rubber gaskets, and LMO electrodes. The side length of the acrylic shell is 55 mm, the internal diameter of the chamber is 36 mm, and the liquid inlet and outlet are funnel-type designs, which is conducive to the passage of solution in and out. The left and right inlets and outlets are connected to the solution and ventilation pipeline, and the capacity of the reaction chamber of the lithium extraction device is 22 mL. The cell is filled with the solution before it is flushed. After flushing, the cell becomes empty, but some residual droplets can still be seen on the shell (Figure 2b).

### 2.2. Electrode Fabrication and Characterization

Firstly, LMO, Super P carbon, and polyvinylidene fluoride (PVDF) are mixed in N-methyl-2-pyrrolidone (NMP) with the mass ratio of 8:1:1. The mixture was coated on a titanium mesh with an effective area of 8 cm^2^ and a pore size number of 50 mesh. Finally, the electrode was dried at 60 °C for 10 h. The morphology of the LMO and the resulting electrodes were characterized by scanning electron microscopy (SEM, TESCAN, MIRA3). The crystal structure was characterized by an X-ray diffractometer (Tongda, TDM-10, Beijing, China).

### 2.3. Electrochemical Characterization

The Cyclic Voltammetry (CV) measurements were carried out with the electrochemical workstation (Chenhua, CHI660E, Shanghai, China) at the scanning rate of 1 mV∙s^−1^. The scan range was 0.4 V~1.0 V. A three-electrode system was used, with an LMO electrode, Ag/AgCl electrode, and platinum plate electrode as the working electrode, reference electrode, and counter electrode, respectively. 1 M LiCl was used as the solution for CV testing.

### 2.4. Lithium Extraction Measurement

The prepared LMO porous titanium mesh electrode and Ag flat plate electrode were used as the anode and cathode, respectively, for lithium-ion extraction. We used a battery testing system (LANHE, M3410A, Wuhan, China) for charging and discharging. The solution conductivity was measured using a flow-through conductivity probe (EDAQ, ET908, Colorado Springs USA). The cation concentration was characterized using an atomic absorption spectrometer (PUXI, TAS-990, Beijing, China). The feed solution for lithium extraction is a simulated Atacama salt lake solution with 40 mM LiCl, 798 mM NaCl and 70 mM MgCl_2_ [33]. Lithium ions were recovered in 20 mM KCl solution. The adsorption process uses a current of 0.25 mA∙cm^−2^ constant current discharge for 0.5 h. The release process uses a current of 0.25 mA∙cm^−2^ constant current charge for 0.5 h. The product purity is calculated as the ratio of lithium ions to the concentration of all cations in the recovered solution.

### 2.5. Modeling and Simulation

**Figure 3:**
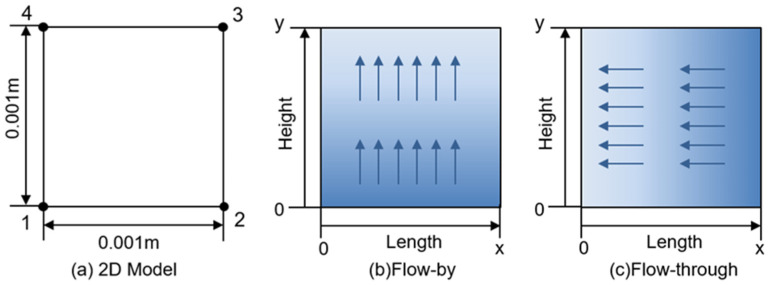
Schematic of the model (**a**) Boundaries and points in 2D model. (**b**) Flow-by model (**c**) Flow-through model.

The motion of lithium ions during discharge can be divided into two parts: from the electrolyte solution to the electrode surface; insertion into the electrode material and transport into the lithium-ion site in the solid phase. Ionic diffusion and conductivity are affected by the properties of electrode materials and internal porosity. In order to more intuitively discuss the influence of flow on lithium ion transport, this model does not consider lithium ion transport in the solid-phase electrode region and focuses on the ion concentration distribution in the boundary region on the electrode surface. The models are two-dimensional and simulated using COMSOL Multiphysics 5.2. Figure 3a. shows the diagram of the model. There are four boundary nodes in the model region. Line segment 14 on the left is the contact boundary between the electrode and the solution, and line segment 23 on the right is the outer boundary of constant concentration.

Diffusion, convection, and electromigration are considered in the model. A three-stage current distribution module was used to solve for the transport of electrolyte species. In the model, the electrode region is not included. We simulated the lithium extraction reaction using electrode surface nodes. In the flow-by model (Figure 3b), the ion concentration at the lower boundary (at y = 0) as well as at the outer boundary (x = L) is set to the initial value, indicating the inflow of the constant concentration source solution from the lower boundary. Adding a flow field along the direction of the electrode surface in the flow-by model. In the flow-through model (Figure 3c), the ion concentration at the outer boundary (x = L) is set to the initial value, indicating the connection to a constant concentration native solution. A flow field perpendicular to the direction of the electrode surface is added. The electrolyte is a multi-ion electron-neutral system containing Li^+^, Na^+^, Mg^2+^, and Cl^−^. However, we only consider the insertion of Li^+^ because the LMO electrode has good selectivity and the insertion of Mg^2+^ and Na^+^ is small. The ion concentration distribution near the electrode surface was determined by solving the Nernst–Planck–Poisson equation. The values of all parameters in this simulation are summarized in Table 1.

## 3. Results and Discussions

### 3.1. Electrode Characterization

**Figure 4:**
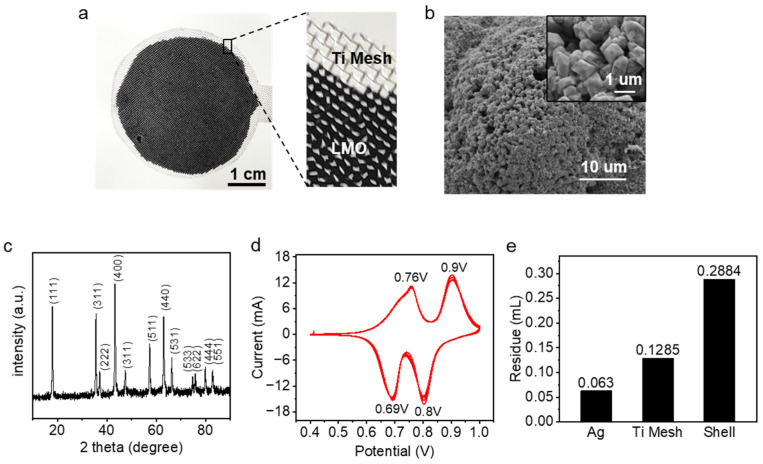
Electrode characterization and residue distribution. (**a**) Porous LMO electrode. (**b**) SEM images of LMO particles. (**c**) XRD patterns of the LMO particles. (**d**) Cyclic Voltammogram of the prepared LMO electrode in 1 M LiCl solution. (**e**) Residual liquid volume distribution in an electrochemical cell.

The porous electrode on the titanium mesh substrate is shown in Figure 4a. Figure 4b shows the SEM image of the LMO particles we used, with a particle diameter of about 1 μm. The XRD spectrum in Figure 4c indicates that the experimentally employed LMO material shows a homogeneous spinel phase, matching the peak of LiMn_2_O_4_ in the standard card (JCPDS 35-0782). Figure 4d shows the CV curves of the obtained electrodes in 1 mM LiCl solution. Two oxidation and reduction peaks appear at 0.76 V, 0.90 V, 0.69 V, and 0.80 V vs. Ag/AgCl, which may correspond to lithium extraction and insertion. After 4 cycles, the positions of the 4 peaks are essentially the same as in the 1st cycle, showing good reversibility of the lithium-ion reaction.

### 3.2. Water Consumption in Gas-Flushed System

Since the volume of the residual solution directly affects the purity of the final product, the distribution of the residual solution inside the device is measured as shown in Figure 4e. There are relatively few residues on the silver and titanium mesh electrodes and more residues on the device housing, with a total residual solution volume of about 0.48 mL. Because of the random nature of the electrode coating uniformity and the random nature of the air washing operation, the residual volume may fluctuate up and down during the operation.

**Figure 5:**
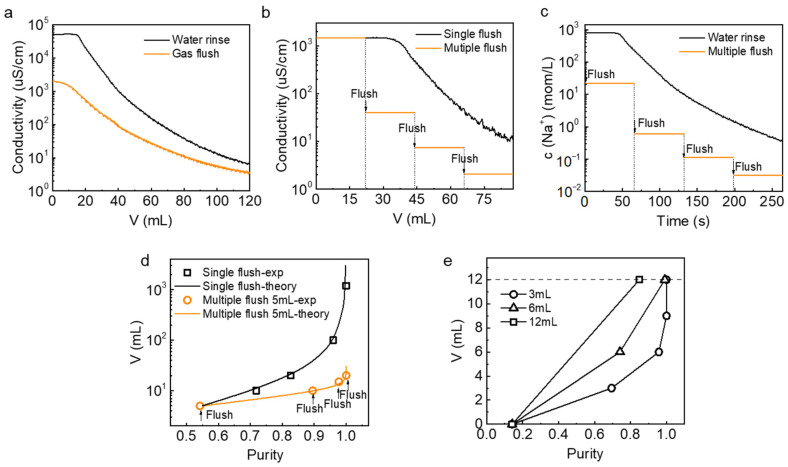
Watersaving performance of the gas flush operation. (**a**) Conductivity changes at the flow outlet in water flush and gas flush mode. (**b**) Conductivity changes at flow outlet in single gas flush and multiple gas flush-22 mL. (**c**) Change of c(Na^+^) at the outlet with operation time. (**d**) Correlation between the purity of recovery solution and consumption of ultrapure water under single and multiple gas flush modes (Experimental and theoretical results). (**e**) Correlation between the purity of recovery solution and consumption of ultrapure water under different multiple gas flush modes.

We first investigated the consumption of ultrapure water during solution exchange for the system without the gas flush operation. After filling the device with simulated salt lake water, ultrapure water was passed into the device at a rate of 20 mL∙min^−1^ after the lithium-ion capture process. Measuring the conductivity at the outlet of the device to characterize the concentration of residual ions. As shown in Figure 5a, the exit conductivity decreases from 52,240 μS∙cm^−1^ in the direct water wash operation mode. In the other group, the conductivity decreases from 2045 μS∙cm^−1^ after the gas flush out the residual liquid in the device and then passing ultrapure water into the device. The conductivity is directly proportional to the concentration of ions in the solution. There are nearly two orders of magnitude differences in the conductivity of the solution in both cases when the ultra-pure water consumption is small, indicating that the concentration of impurity ions remaining after the gas flush is lower. The use of gas flush operation can greatly reduce the amount of ion residue in the source solution. However, the single gas flush operation reduces the concentration of the residual solution only in the initial stage. The reduction of conductivity gradually becomes slower with the continuous passage of ultrapure water. In the latter part of the conductivity curve, the conductivity at the outlet of the single gas flush and water rinse is becoming closer and closer, gradually decreasing to about 10 μS/cm. However, this is still far from the ultra-pure water conductivity of 0.17 μS/cm to meet the requirements of passing through the recovery solution for further treatment. Continuous rinsing with ultra-pure water is still required to reduce the amount of residue and avoid the impact on the purity. In order to further reduce water consumption and save operation time, we considered the use of multiple gas flush to achieve a relatively small amount of residue.

The multiple gas flush operation is that after gas flushing the source solution, a small amount of ultrapure water is passed into the device to clean the device housing and the residue on the electrode surface, then gas flushing out the waste solution and repeating the process. As shown in Figure 5b for the stepped line segment, each arrow indicates 22 mL of ultra-pure water, followed by a nitrogen flush. After the second gas wash, the electrical conductivity at the outlet sharply decreased from 1472.2 μS/cm to 40.55 μS/cm, 7.458 μS/cm after the third gas wash, and 2.101 μS/cm after the fourth gas wash. The electrical conductivity showed a step-down trend. However, with the same water consumption, the conductivity showed a slow downward trend and finally dropped to about 10 μS/cm after a single gas flush with continuous water washing. For the same water consumption, compared with a single gas flushing, the multiple gas flush approach is easier to obtain lower conductivity, that is, to reduce more residue of impurities. Figure 5c represents the Na^+^ ion concentration at the outlet of the device as a function of time. The results show that under the same pumping velocity, multiple gas flush approaches take less time to achieve the same low sodium ion concentration. The flush time is negligible compared to the time it takes to pump in the solution, which is a huge advantage of the gas flushing operation.

Within a complete charge/discharge process (Figure S1), the purity of the recovery solution was measured to compare the water consumption during the conversion of the solution in different gas flush modes. In Figure 5d, each point of the 5 mL-gas flush represents the purity of the recovery solution after one time of gas flush followed by ultra-pure water cleaning. After four times gas flushing with 5 mL of ultrapure water, the water consumption is 20 mL, and the purity of the recovery solution is 100%. Single gas flush points represent the purity of the recovery solution without repeatedly passing ultrapure water, but directly passing different volumes of ultrapure water for cleaning. However, after consuming 20 mL of ultrapure water in a single gas flushing, the purity of the recovery solution was only 82%. The experimentally measured purity results match the water consumption curve (Figure 5d) obtained from theoretical calculations based on the actual residual and source solution ion concentrations. Details of the calculation can be found in Supplementary Materials (Table S1, Equations (S1)–(S5)). The black theoretical curve in Figure 5d represents a near-exponential increase in water consumption in the operation of a single gas flush. The closer the purity of the recovery solution is to 100%, the faster the water consumption increases. The purity was 71.83% at 10 mL of water consumption and 95.92% at 100 mL of water consumption. In order to achieve 100% purity of the recovered liquid, a single gas flush needs to consume 1200 mL of ultra-pure water, which is 60 times the water consumption of a 5 mL multiple gas flush. The results show that multiple gas flush operation saves significantly on ultrapure water compared to single gas flush, and only a small amount of water is consumed to achieve high purity recovery solution.

In order to further investigate the specific operation mode of multiple gas flush, two times 6 mL-gas flush and four times 3 mL-gas flush were compared at a total water consumption of 12 mL. As shown in Figure 5e, the lower the water consumption of each gas flush, the higher the purity for the same total water consumption. It can also be proved by theoretical calculation (Figure S2). However, it is necessary to consider the complexity of the whole operation process, and the number of repetitions of gas flush should not be too many.

### 3.3. Simulation in Different Flow Modes

**Figure 6:**
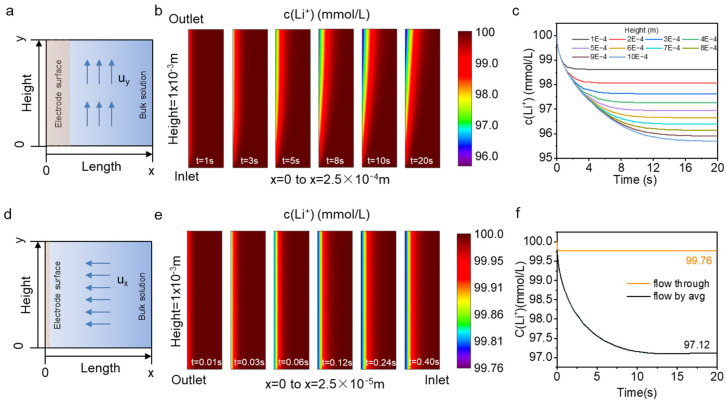
Simulation results (**a**) flow-by model (**b**) Li^+^ concentration distribution from x = 0 to 2.5 × 10^−4^ m in flow-by model (**c**) The concentration changes of Li^+^ on x = 0 at different heights in flow-by mode (**d**) flow-through model (**e**) Li^+^ concentration distribution from x = 0 to 2.5 × 10^−5^ m in flow-through model (**f**) The average concentration changes of Li^+^ on x = 0 at different flow mode.

The closer to the electrode surface the lower the lithium-ion concentration is (Figure S3). This is due to the insertion of lithium ions into the electrode material during the discharge process, while diffusion and convection will replenish the lithium ions on the electrode surface. Eventually, a balance is reached between the replenishment rate of lithium ions and depletion rates, forming a depletion zone [27]. Set the flow rate u = 0.1 mm·s^−1^, the reaction local current density i_loc_ = 2.5 A·m^−2^. The transient state of the whole model was studied at 0.01 s intervals. Figure 6a shows the flow-by model, where the solution flows in the direction along the electrode surface. Figure 6b intercepts the lithium-ion concentration distribution in the x-direction from 0 to 2.5 × 10^−4^ m. The lithium-ion concentration on the electrode surface is not uniformly distributed along the y-direction and shows a decreasing trend along the flow direction. As time increases, the lithium concentration on the electrode surface gradually decreases until it reaches equilibrium. Figure 6c shows the variation of lithium concentration with time at the electrode surface (x = 0) at points of different heights y. The lithium-ion concentration is lowest at the exit at the upper edge of the electrode surface (y = 1 × 10^−3^ m), reaching an equilibrium concentration of 95.7 mM at about t = 20 s. The flow-through model is shown in Figure 6d, and the flow direction of the source solution in this flow pattern is perpendicular to the electrode surface and directly across the electrode. Figure 6e intercepts the concentration distribution in the x-direction from 0 to 2.5 × 10^−5^ m. The depletion zone length of flow-through is an order of magnitude smaller compared to the flow-by model. The lithium-ion concentration at the electrode surface in the flow-through model is uniformly distributed along the y-direction and reaches a stable value of concentration in a short time (t = 0.18 s).

Due to the non-uniform concentration distribution on the flow by surface, the average concentration curve with time for the entire electrode surface (x = 0, from y = 0 to y = 1 × 10^−3^ m) in this mode was integrated (Figure 6f). The initial lithium-ion concentration on the electrode surface is 100 mM, and the average lithium-ion concentration after reaching a steady state is 97.12 mM. Compared with the flow-by model, the flow-through model reaches stability faster and the lithium-ion concentration at x = 0 on the electrode surface is 99.76 mM, which is much higher than 97.12 mM. In our simulation, the concentration of lithium ions at the electrode surface is higher in the flow-through mode under the same flow and discharge conditions. The magnitude of the lithium insertion reaction current in the electrode is related to the lithium-ion concentration at the electrode surface. Higher lithium concentration leads to faster lithium insertion and a faster mass transfer rate at the solid–liquid interface. Further, higher concentrations are less likely to lead to lithium-ion depletion, which can increase the yield. For electrodes with insufficient selectivity, if the electrode surface lithium ion concentration is low, other cations may insert into the electrode leading to lower purity of the recovery solution. Therefore, in electrochemical lithium extraction systems, better lithium extraction performance can be obtained by selecting flow-through reactors that can maintain higher lithium ion concentrations on the electrode surface.

### 3.4. Lithium Extraction Performance of the System in Simulated Salt Lake Water

**Figure 7:**
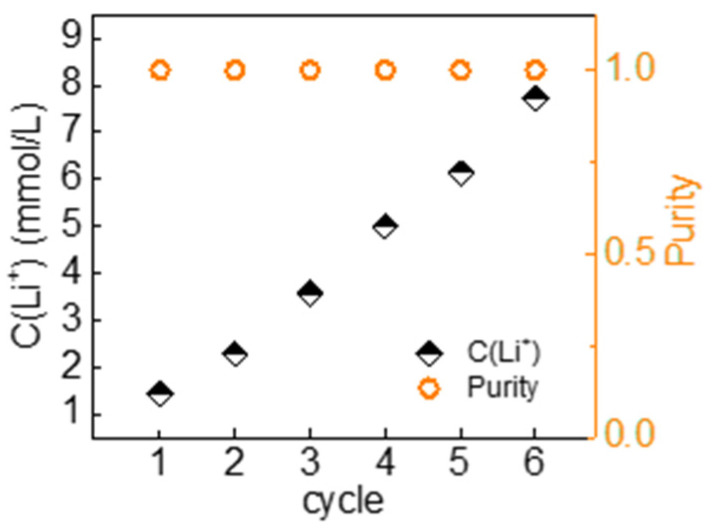
Purity and Li^+^ concentration in the recovery solution extracted with gas flushing system from Atacama simulated water.

Considering the complexity of the whole operation process, we used 6 mL-gas flush twice as the operation mode of the cycle to verify the lithium extraction performance of the whole system. After six cycles, the purity of the recovery solution was maintained close to 100% (Figure 7), and the lithium-ion concentration was enriched to 7.7 mM with an average yield of 1.54 mmol∙g^−1^∙h^−1^ and an average energy consumption of 0.732 kWh∙kg^−1^ (Table 2). The system operated with good performance and obtain a high-purity recovery solution with very low energy consumption. The concentration of the recovery solution can be further improved by some operational optimization, such as increasing the reaction current density, reducing the volume of the recovery solution, and using electrode materials with higher lithium capacity, etc. Although studies have proved that LMO has good selectivity and stability, more cycling tests should be carried out in the system to explore capacity attenuation during the lithium extraction process in the future.

## 4. Conclusions

In summary, we propose an electrochemical lithium extraction with gas flushing of porous electrodes. The system can operate continuously and automatically. We demonstrate that the gas flush operation can significantly save the consumption of ultrapure water during the electrochemical lithium extraction, and the multiple gas flush operation consumes less water and save time. The water consumption of multiple gas flush operations obtains a purity close to 100% in a recovery solution is 20 mL, which is only 1/60 of that of a normal single flush. We simulate the concentration distribution of lithium ions on the electrode surface in both flow-through and flow-by modes and demonstrate that flow-through significantly reduces the effect of medium concentration polarization and maintains a high lithium ion concentration value. This work also verifies the lithium extraction performance of the whole system by using Atacama simulated salt lake water as the source solution, which achieves lithium extraction with a purity close to 100% and an average energy consumption of 0.732 kWh∙kg^−1^ in each cycle. These results provide a feasible method for the large-scale operation of electrochemical lithium extraction from seawater/brine.

## Figures and Tables

**Table 1:** Parameters used in the simulation [34].

Parameter	Description	Value
L	Thickness of depletion region [m]	1 × 10^−3^
H	Length of electrode surface [m]	1 × 10^−3^
λ_S_	Thickness of stern layer [m]	3 × 10^−10^
D_Li+_	Diffusion coefficient of Li^+^ [m^2^ s^−1^]	9.9 × 10^−10^
D_Na+_	Diffusion coefficient of Na^+^ [m^2^ s^−1^]	13.4 × 10^−10^
D_Mg2+_	Diffusion coefficient of Mg^2+^ [m^2^ s^−1^]	7.5 × 10^−10^
D_Cl−_	Diffusion coefficient of Cl^−^ [m^2^ s^−1^]	1.98 × 10^−9^
c_Li+,0_	Initial concentration of Li^+^ in electrolyte [mol m^−3^]	100
c_Na+,0_	Initial concentration of Na^+^ in electrolyte [mol m^−3^]	100
c_Mg2+,0_	Initial concentration of Mg^2+^ in electrolyte [mol m^−3^]	100
c_Cl−,0_	Initial concentration of Cl^−^ in electrolyte [mol m^−3^]	400
ε	Permittivity of the electrolyte [F m^−1^]	78.5
T	Absolute temperature [K]	298.15
F	Faraday constant [C mol^−1^]	96,484
R	Molar gas constant [J mol^−1^ K^−1^]	8.314

**Table 2:** Energy consumption and yield performance in Continuous operation.

Cycle	Production (mmol⋅g^−1^⋅h^−1^)	Energy Consumption (kWh⋅kg^−1^)
1	1.714	0.476
2	1.029	0.677
3	1.543	0.600
4	1.714	0.904
5	1.371	0.955
6	1.886	0.781
Average	1.543	0.732

## Data Availability

The data presented in this study are available on request.

## Supplementary Materials

The following supporting information can be downloaded at: https://www.mdpi.com/article/10.3390/nano13091471/s1, Figure S1: Electrochemical properties of gas flushing system in simulated Atacama salt lake simulated solution; Figure S2: Theoretical calculation of the total water consumption and purity of multiple gasflush with different ultrapure water volumes per pass; Figure S3: The distribution of Li^+^ concentration along the x-direction in the model; Table S1. Parameters used in the water consumption calculation.
