# Supplementary material for: Electrochemical Lithium Extraction with Gas Flushing of Porous Electrodes
Source: Nanomaterials (Basel). 2023 Apr 26;13(9):1471. doi: 10.3390/nano13091471 (PMC10179911; doi:10.3390/nano13091471)
Supplement: Supplementary file 1 [file nanomaterials-13-01471-s001.zip › nanomaterials-2310130-supplementary.pdf]

nanomaterials

Supplementary Information

Electrochemical Lithium Extraction with Gas Flushing of Porous electrodes

Shengyao Wang, Xuyu Yu and Xuejiao Hu *

MOE Key Laboratory of Hydraulic Machinery Transients, School of Power and Mechanical Engineering, Wuhan University, Wuhan 430072, China; wangshengyao@whu.edu.cn (S.W)
* Correspondence: xjhu@whu.edu.cn

Supplementary Note

We have made theoretical calculations to predict the relationship between the water consumption of gas flush and the purity of the recovery solution. The following parameters are included in the complete lithium extraction process.

Table S1. Parameters used in the water consumption calculation.

Parameters	Description
V_0	Volume of source solution passed in [mL]
V_c^1、 $V_c^2 \ldots$ V_c^n	Volume of residual solution in the device after each gas flush [mL]
V_{up}^1、 $V_{up}^2 \ldots$ V_{up}^n	Volume of ultrapure water passed in after gas flush [mL]
$C_0(Li^+)$	Concentration of Li^+ in the source solution [mol m^{-3}]
$C_0(Mg^{2+})$	Concentration of Mg^{2+} in the source solution [mol m^{-3}]
$C_0(Na^+)$	Concentration of Na^+ in the source solution [mol m^{-3}]
$\eta_{(Li^+)}$	Selectivity coefficient of electrodes for Li^+
$\eta_{(Mg^{2+})}$	Selectivity coefficient of electrodes for Mg^{2+}
$\eta_{(Na^+)}$	Selectivity coefficient of electrodes for Na^+
m	Number of repeated gas flush
C	Capacity of the discharge process [mAh]
K	Conversion constant [mmol mAh^{-1}]

After the discharging process, the concentration of lithium ions in the remaining solution is:

$$C_s(Li^+) = \frac{V_0 \cdot C_{0(Li^+)} - X \cdot K \cdot \eta_{(Li^+)}}{V_0} \tag{S1}$$

After gas flushing out the brine, the amount of lithium ions remaining in the device is:

$$n_{c_0}(Li^+) = C_s(Li^+) \cdot V_c \tag{S2}$$

After repeating the gas flush for m times, the amount of Li^+ in the residual liquid is:

$$n_c^m(Li^+) = n_{c_0}(Li^+) \cdot \left(\frac{V_c}{V_c + V_{up}}\right)^m \tag{S3}$$

Assumeing that the charging process has no side reactions other than ion intercalation. After passing the recovery solution in the device, the amount of lithium ions in the recovery solution is:

$$n_j(Li^+) = X \cdot K \cdot \eta_{(Li^+)} + n_c^m(Li^+) \tag{S4}$$

Similarly, the amount of sodium ions $n_j(Na^+)$ and magnesium ions $n_j(Mg^{2+})$ in the recovery solution can be calculated.

The purity of the recovery solution after each gas flush can be calculated by stepwise iterations (from Equation S1 to Equation S4) given the initial conditions. Purity of the recov-ery solution is:

$$Purity = \frac{n_j\ (Li^+)}{n_j(Li^+) + n_j(Na^+) + n_j(Mg^{2+})} \quad (S5)$$

We use theoretical calculations to predict which gas flush mode is more convenient and water-saving.

Supplementary Figures

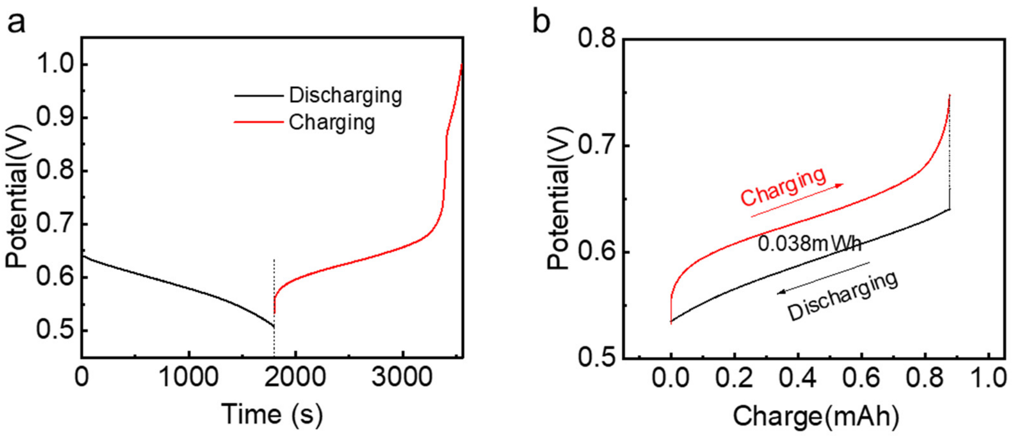

Figure S1. Electrochemical properties of gas flushing system in simulated Atacama salt lake simulated solution. (a) Variation of potential with time during charge-discharge process. (b) Energy consumption of lithium extraction.

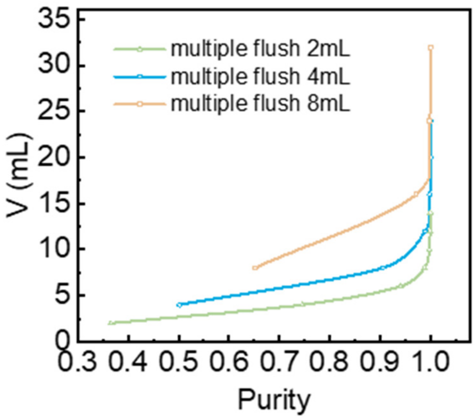

Figure S2. Theoretical calculation of the total water consumption and purity of multiple gasflush with different ultrapure water volumes per pass.

The data in Figure S2 are calculated based on the ion concentration of Atacama salt lake water. The initial conditions and charging and discharging conditions are referred to the experimental settings. The lower the water consumption per gas wash, the lower the total water consumption when reaching high purity.

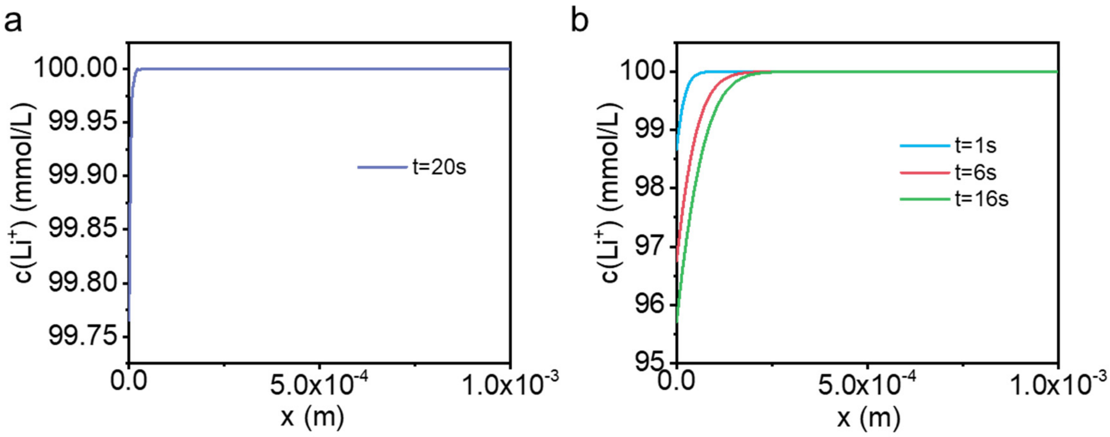

Figure S3. The distribution of Li^+ concentration along the x-direction in the model. (a) In the flow-through model, t = 20s. (b) At the flow outlet(y=H) of the flow-by model.
